# Effectiveness of Nirsevimab Immunoprophylaxis Administered at Birth to Prevent Infant Hospitalisation for Respiratory Syncytial Virus Infection: A Population-Based Cohort Study

**DOI:** 10.3390/vaccines12040383

**Published:** 2024-04-04

**Authors:** Guillermo Ezpeleta, Ana Navascués, Natividad Viguria, Mercedes Herranz-Aguirre, Sergio Enrique Juan Belloc, Juan Gimeno Ballester, Juan Carlos Muruzábal, Manuel García-Cenoz, Camino Trobajo-Sanmartín, Aitziber Echeverria, Iván Martínez-Baz, Noelia Vera-Punzano, Itziar Casado, Héctor López-Mendoza, Carmen Ezpeleta, Jesús Castilla

**Affiliations:** 1 Instituto de Salud Pública de Navarra, 31003 Pamplona, Spain; guillermo.ezpeleta.lobato@navarra.es (G.E.); imartinba@navarra.es (I.M.-B.); hlopezmg@navarra.es (H.L.-M.); 2 Clinical Microbiology Department, Hospital Universitario de Navarra, 31008 Pamplona, Spain; 3 Instituto de Investigación Sanitaria de Navarra (IdiSNA), 31008 Pamplona, Spainmercedes.herranz.aguirre@navarra.es (M.H.-A.); 4 Paediatrics Department, Hospital Universitario de Navarra, 31008 Pamplona, Spain; 5 Paediatrics Department, Hospital Reina Sofía, 31500 Tudela, Spain; 6 Paediatrics Department, Hospital García Orcoyen, 31200 Estella, Spain; 7 Gynecology and Obstetrics Department, Hospital Universitario de Navarra, 31008 Pamplona, Spain; 8 CIBER Epidemiología y Salud Pública (CIBERESP), 31003 Pamplona, Spain

**Keywords:** respiratory syncytial virus, infants, nirsevimab, monoclonal antibodies, hospitalisation, vaccination effectiveness, vaccination impact

## Abstract

Respiratory syncytial virus (RSV) infection is a frequent cause of hospitalisation in the first few months of life; however, this risk rapidly decreases with age. Nirsevimab immunoprophylaxis was approved in the European Union for the prevention of RSV-associated lower respiratory tract disease in infants during their first RSV season. We evaluated the effectiveness of nirsevimab in preventing hospitalisations for confirmed RSV infection and the impact of a strategy of immunisation at birth. A population-based cohort study was performed in Navarre, Spain, where nirsevimab was offered at birth to all children born from October to December 2023. Cox regression was used to estimate the hazard ratio of hospitalisation for PCR-confirmed RSV infection between infants who received and did not receive nirsevimab. Of 1177 infants studied, 1083 (92.0%) received nirsevimab. The risk of hospitalisation for RSV was 8.5% (8/94) among non-immunised infants versus 0.7% (8/1083) in those that were immunised. The estimated effectiveness of nirsevimab was 88.7% (95% confidence interval, 69.6–95.8). Immunisation at birth of infants born between October and December 2023 prevented one hospitalisation for every 15.3 immunised infants. Immunisation of children born from September to January might prevent 77.5% of preventable hospitalisations for RSV in infants born in 2023–2024. These results support the recommendation of nirsevimab immunisation at birth to children born during the RSV epidemic or in the months immediately before to prevent severe RSV infections and alleviate the overload of paediatric hospital resources.

## 1. Introduction

Respiratory syncytial virus (RSV) is a leading cause of bronchiolitis and lower respiratory tract disease in infants [1]. RSV infection is a frequent cause of hospitalisation in the first few months of life. The risk of RSV-related hospital admission in infants decreases rapidly with increasing months of age [2,3,4,5,6].

A long-acting monoclonal antibody, named nirsevimab (Beyfortus^®^, AstraZeneca/Sanofi Pasteur), has been developed for the prevention of RSV-associated lower respiratory tract disease in infants with high-risk conditions [7,8]. The successful results of efficacy and safety of nirsevimab in infants with high-risk conditions lead us to consider the potential benefit for healthy infants during their first RSV season [9,10,11,12,13]. Two clinical trials evaluated the efficacy of nirsevimab to prevent RSV-associated lower respiratory tract disease in healthy infants, reporting estimates between 76.4% and 83.2% depending on the outcome evaluated (medically attended RSV infection, hospitalisation, or severe cases) [8,9]. A mathematical model suggested the benefit of immunisation for infants experiencing their first RSV season [14]. Supported by this evidence, on 4 November 2022, the European Commission approved the commercialisation of nirsevimab [6]. Nevertheless, several questions remained pendent, such as the effectiveness of nirsevimab in real-life conditions, the risk–benefit balance of a universal immunisation strategy, and the search for the best strategy for infant immunisation to achieve high immunisation coverage and a good cost–benefit balance. The high price of nirsevimab (>200 EUR/infant) may be a limitation for extending the immunisation to healthy infants.

Although two subgroups of RSV (A and B) circulate over time, differences in the effectiveness of nirsevimab against each subgroup have not been described, and no other genetic variability has demonstrated a relevant capacity to evade the effect of this monoclonal antibody.

The risk of severe RSV outcomes is usually concentrated in a few weeks of the year, following a seasonal pattern that depends on the region [15], but some variability has been described during the COVID-19 pandemic [16]. In Spain, RSV epidemics usually peak in December or early January, and, on average, infants born between October and December account for more than half of the RSV-related hospitalisations of children younger than one year [5,6]. The knowledge of this epidemiological pattern allowed us to search for efficient strategies for using nirsevimab.

The present study had three objectives: (1) to evaluate the effectiveness of nirsevimab immunoprophylaxis at birth in preventing hospitalisations for confirmed RSV infection in infants, (2) to assess the impact of an immunisation strategy of newborn children at maternity wards during the RSV season, and (3) to estimate the potential impact of other strategies that extend the immunisation to additional birth cohorts.

## 2. Materials and Methods

### 2.1. Design

This population-based study was performed in the region of Navarre (~670,000 inhabitants and ~4750 births annually), Spain. The study design consisted of three parts: (1) a descriptive analysis of data from the enhanced epidemiological surveillance system and the immunisation register, (2) a prospective cohort design to evaluate the effectiveness of nirsevimab in infants, and (3) the integration of results from the previous parts to assess the impact of this intervention and to compare it with the potential impact of other immunisation strategies.

### 2.2. Setting, Information Sources, and Variables

In July 2023, the Spanish Ministry of Health recommended immunising infants with nirsevimab, prioritised as follows: (1) infants at high risk of developing severe disease from RSV infection (preterm children < 35 weeks gestational age during their first RSV season and infants aged less than 24 months with conditions such as congenital heart disease with significant hemodynamic involvement or bronchopulmonary dysplasia); and (2) infants under six months of age born from April 2023 to March 2024, prioritising those born during the RSV season [5]. A sufficient number of doses of nirsevimab for prospective administration to all newborn children at births were available from the end of September 2023.

In Navarre, nirsevimab immunoprophylaxis was publicly funded and prospectively offered at birth in maternity wards to all children born between October 2023 and January 2024. Nirsevimab was also offered to infants residing in Navarre who were born abroad during the mentioned period. A dose of 50 mg was administered intramuscularly for infants with body weight < 5 kg and a 100 mg dose for infants with body weight ≥ 5 kg. All immunised infants and those non-immunised due to parents’ refusal were registered in the Regional Immunisation Register. The immunisation was considered potentially effective one day after administration.

All infants admitted to the hospital due to acute respiratory illness were routinely tested by a polymerase chain reaction (PCR) test for RSV, and all positive results were electronically reported to the enhanced epidemiological and virological surveillance system. As part of the epidemiological surveillance, medical doctors reviewed all hospitalised infants with laboratory-confirmed RSV, and only those admissions due to RSV-associated lower respiratory tract disease were considered. Infant patients attended at the emergency room who did not require hospitalisation were tested by PCR for RSV according to the paediatrician’s criteria.

Infants born in 2023 and January 2024 were detected from administrative databases with their respective identification code number, date of birth, sex, and residence region. The individual identification code was used to link administrative data, information on cases detected by the enhanced epidemiological and virological surveillance of RSV and the nirsevimab immunisations from the regional register.

### 2.3. Statistical Analysis

The weekly number of hospitalisations due to confirmed RSV infection in infants born in 2023 and 2024, by birth-month, was obtained from the epidemiological surveillance.

Nirsevimab effectiveness was evaluated within a prospective cohort of all infants born in Navarre from October to December 2023 (birth cohorts included in the intervention and with RSV-confirmed cases) who continued residing in the region. Infants born in January 2024 were omitted because none were hospitalised for RSV. The follow-up ended on 28 January 2024, after two consecutive weeks without hospital admissions for RSV infection in the cohort population and because there was no other hospitalisation in the eight following weeks. The follow-up of the included infants began on the date of birth and ended on 28 January 2024 or the date of the first confirmed RSV specimen, whichever occurred first. The percentage of hospitalisations for confirmed RSV infection in infants was calculated by nirsevimab immunisation status.

The hospitalisation rates for confirmed RSV infection were compared between infants who received nirsevimab and those who did not using Cox regression models to obtain hazard ratios (HR) with their 95% confidence intervals (CI). The number of calendar days since 1 October 2023 was used as the underlying time scale, with entry time being the date of birth. Immunisation status with nirsevimab was defined as a time-dependent variable. Person days at risk were used as the denominator for incidence rates. Models were adjusted for sex and week of birth. The effectiveness was estimated as (1 − HR) × 100.

Other outcomes evaluated were confirmed RSV infection attended in an emergency room regardless of the hospital admission and admission to the intensive care unit (ICU).

A sensitivity analysis included infants born from 1 October 2023 to 31 January 2024 and extended the follow-up from 1 October 2023 to 25 February 2024. Infants born in January 2024 were included in the nirsevimab recommendation but were not included in the main analysis because no RSV-confirmed cases were detected.

The cases recorded by epidemiological surveillance and estimates of nirsevimab effectiveness were used to estimate the number of hospitalisations prevented by dividing the number of hospitalisations observed in immunised infants by the HR of RSV-related hospitalisation. The number of infants who need to be immunised with nirsevimab to prevent one RSV-related hospitalisation was calculated as the ratio of the number of immunoprophylaxis administered and the estimated number of hospitalisations prevented. The monthly number of births registered in the region was used as the denominator to calculate rates.

The potential impact of extending the immunisation to infants born before October was estimated by applying the estimates of immunisation coverage and effectiveness to the monthly number of births. For September, we assumed immunisation of newborn children at maternity wards with similar immunisation coverage to that observed in October–December. For August and previous months, we considered a catch-up strategy with an immunisation coverage of 81.4%, as was reported in the primary healthcare catch-up in another Spanish region [17].

## 3. Results

### 3.1. Detections and Hospitalisations for RSV in the 2023–2024 Season

**Figure 1:**
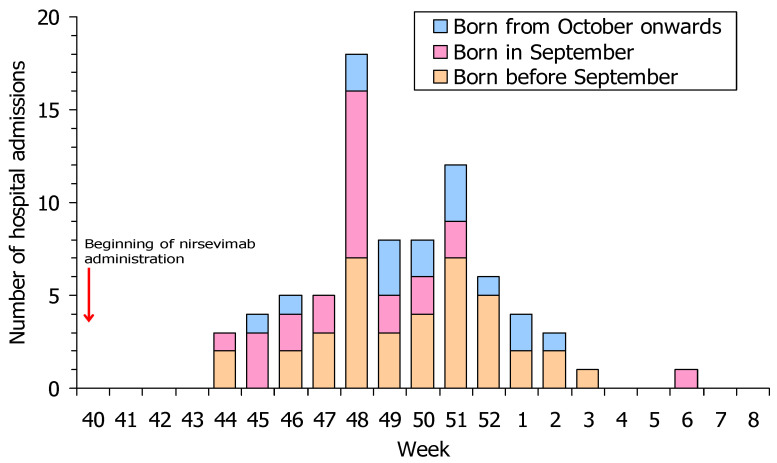
Weekly number of hospitalisations for confirmed respiratory syncytial virus infection during the 2023–2024 season among infants born in Navarre in 2023 and 2024 by birth month.

The enhanced epidemiological and virological surveillance of RSV cases in Navarre showed an early season with continuous RSV detections in infants from 10 October 2023 to 15 February 2024. In infants born in 2023 and 2024, there were 78 hospitalisations due to RSV, which were distributed from 1 November 2023 to 8 February 2024 (weeks 44/2023 to 6/2024), and the highest numbers of admissions were registered between weeks 48 and 51 (27 November to 24 December 2023). Of these hospitalised infants, 16 (20.5%) were born from October onwards (target population for immunisation), 30.8% were born in September, 37.2% from April to August, and 11.5% from January to March 2023. There were no RSV-related hospitalisations in infants born in 2024 (Figure 1).

### 3.2. Nirsevimab Effectiveness in Preventing RSV-Related Hospitalisation

Of 1186 infants born in Navarre from October to December 2023, 9 were excluded from the study because they resided outside the region. Of 1177 infants included in the cohort study, 1083 (92.0%) received nirsevimab, 95.6% of them within seven days after birth. No adverse effects were reported.

**Figure 2:**
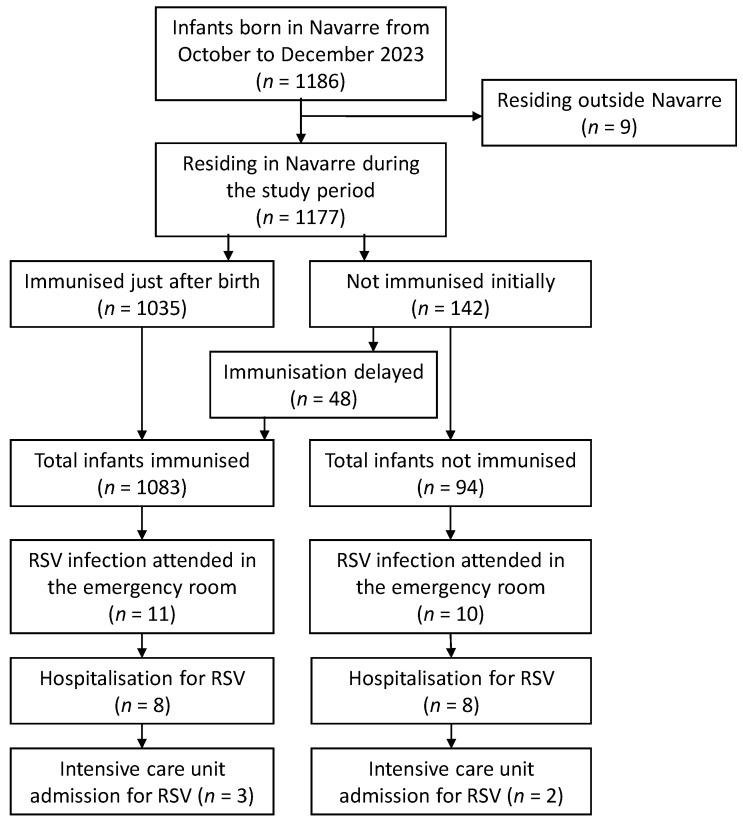
Flow chart of the population cohort of infants born from October to December 2023.

Up to 28 January 2024, 21 infants attended the emergency room due to confirmed RSV infection. Sixteen of them were hospitalised due to RSV infection (all of them with a diagnosis of bronchiolitis) and five were admitted to the ICU (Figure 2). The first hospitalisation for RSV was on 11 November and the last was on 10 January, covering a maximum follow-up of 101 days since the beginning of the administration of nirsevimab.

The 16 hospitalised cases had a median age of 38.5 days (a range of 14 to 60 days), and 8 (50.0%) had received nirsevimab immunoprophylaxis, all within the first three days of life. No infant hospitalised for RSV infection had a high-risk condition diagnosis prior to hospital admission, but one immunised infant was diagnosed with metabolopathy after the RSV diagnosis.

The risk of RSV-related hospitalisation was 8.5% (8/94) among non-immunised infants and 0.7% (8/1083) among those that were immunised. The risks of ICU admission were 2.1% (2/94) and 0.3% (3/1083), respectively (Figure 2, Table 1).

**Figure 3:**
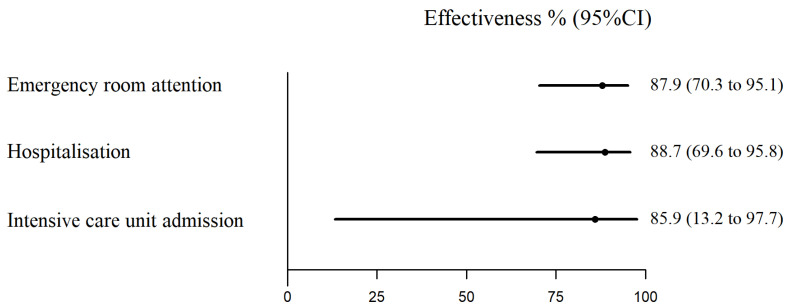
Effectiveness of nirsevimab in preventing laboratory-confirmed respiratory syncytial virus cases.

The estimate of the immunoprophylaxis effectiveness in preventing hospitalisation for lower respiratory tract infection due to RSV was 88.7% (95% CI, 69.6–95.8). No significant differences were observed in the effectiveness by sex or birth month against hospitalisations. Effectiveness estimates were similarly high in preventing emergency room consultations (87.9%; 95% CI, 70.3–95.1) or cases requiring ICU admission (85.9%; 95% CI, 13.2–97.7) (Table 1 and Figure 3).

A sensitivity analysis included all children born from 1 October 2023 to 31 January 2024, and the follow-up period was extended until 25 February 2024. Estimates for nirsevimab effectiveness in preventing RSV-related hospitalisation and ICU admissions remained unchanged. The effectiveness estimate in preventing emergency-room consultations increased slightly (89.0%; 95% CI, 73.7–95.4) due to the inclusion of an additional non-immunised case.

### 3.3. Evaluation of the Immunisation Strategy

Among infants born from October to December, 16 hospitalisations due to RSV were observed up to 14 January 2024 (13.6 per 1000 infants). As nirsevimab immunoprophylaxis prevented an estimated number of 60.2 hospitalisations per 1000 infants, the rate of RSV-related hospitalisation would have been 73.7 per 1000 infants born from October to December in the absence of immunisation. The intervention focused on infants born between October and December 2023 and prevented 47.6% of the RSV-related hospitalisations in infants born during 2023 in Navarre that would have occurred without immunisation.

**Figure 4:**
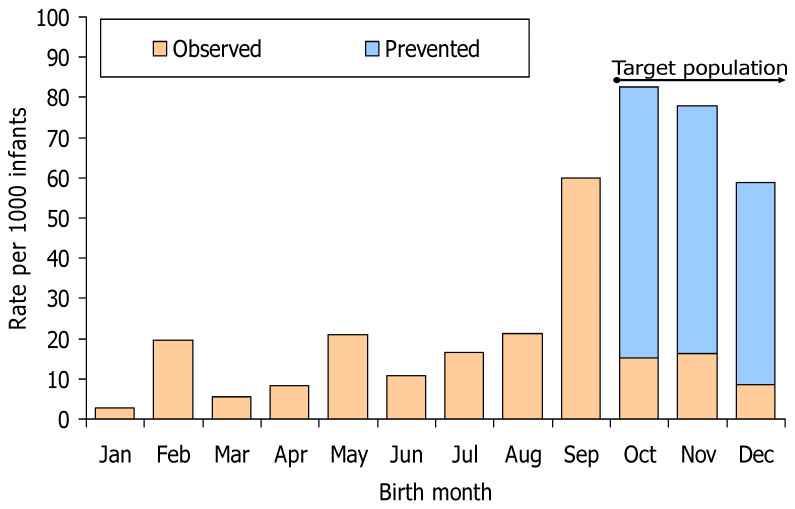
Observed incidence of hospitalisation for confirmed respiratory syncytial virus infection and estimated incidence of hospitalisations prevented by birth month among children born in Navarre in 2023 and follow-up from October 2023 to 14 January 2024.

The incidence was higher in infants born in September 2023 (60 cases per 1000 infants), who were not included in the prospective immunisation strategy (Figure 4). However, assuming a similar immunisation coverage and effectiveness to that observed in the immunisation of newborn children, nirsevimab immunoprophylaxis could have prevented 49.0 hospitalisations per 1000 infants born in September.

The rates of RSV-related hospitalisations observed and those preventable with nirsevimab were lower in the cohorts of infants born in the April–August period, 15.7 and 11.3 per 1000 infants, respectively.

An estimate of hospitalisations prevented by nirsevimab in children born in January 2024 could not be obtained because there were no RSV-related hospitalisations in infants born in 2024 due to the very low RSV circulation in the population. As a conservative assumption, we assigned an estimated number of four hospitalisations prevented by nirsevimab immunoprophylaxis among infants born in January 2024.

Immunisation of children born from September 2023 to January 2024 would have prevented 77.5% (95.4/121.5) of preventable hospitalisations for RSV in infants born in 2023–2024 and 81.8% (94.4/115.3) of those preventable with a strategy that combined prospective immunisation of children born from September to January and the catch-up of those born from April to August.

Only 15.3 infants born from October to December 2023 needed nirsevimab administration to prevent one RSV-related hospitalisation. This parameter remained low (18.8) for children born in September 2023 and increased to 71.7 for children born from April to August 2023 (Table 2).

## 4. Discussion

The results of the present study show that nirsevimab immunoprophylaxis at birth was highly effective (88.7%) at preventing hospitalisation due to RSV-associated lower respiratory tract disease in children who were born during the RSV epidemic or in the immediate previous months. The effectiveness was similarly high with regard to preventing RSV infection attended in the emergency room (87.9%) and those severe cases admitted to the ICU (85.9%).

The MELODY clinical trial in healthy infants reported an efficacy of 76.4% in preventing medically attended RSV infection, 76.8% against hospitalisation, and 78.6% against severe cases [9,10]. The HARMONIE trial found an efficacy of 83.2% in preventing RSV-related hospitalisation and 75.7% against severe cases [11]. Early estimates of the nirsevimab effectiveness from an observational study in Spain suggest an effectiveness of 70.2% or 84.4%, depending on the method used [18]. The introduction of nirsevimab prophylaxis has been related to a decreased incidence of severe RSV infections in Luxembourg [19]. The estimates from the present study were consistent with those reported in the United States [20]. However, they were slightly higher than those reported in clinical trials and previous observational studies, which might be explained by the fact that infants were immunised a few weeks before or during a period of high RSV circulation, and, therefore, the antibody titres at virus exposure were expected to be high.

The lack of reported adverse effects is consistent with other studies and supports the safety of this immunoprophylaxis [9,11,21,22].

In Navarre, nirsevimab was offered at birth in maternity wards, so infants were protected from their first few days of life. Immunisation in primary healthcare implies assuming the possible risk of infection in the first few days of life until the nirsevimab is administratered. The immunisation coverage in this study was very high (92.0%), similar to that described in another Spanish region in newborn infants immunised in hospitals (92.6%), but higher than the coverage in the catch-up group immunised in primary healthcare (81.4%) [17].

The immunisation strategy at birth in Navarre focused on children born in the four months (October to January) with the highest rates of hospitalisation for RSV in pre-pandemic seasons [6]. This strategy made it possible to achieve a high impact of this intervention. Immunisation at birth of children born from October to December 2023 was a very efficient intervention since one RSV-related hospitalisation was prevented with only 15.3 children immunised. Immunisation was offered at birth in maternity wards, which reduced the number of administration points and the time from childbirth to immunisation, contributing to the increase in immunisation coverage. The prevention of a notable proportion of hospitalisations for RSV in infants during the RSV epidemic contributed to paediatric hospital resources not being overwhelmed.

The nirsevimab immunoprophylaxis was offered at birth to children born since 1 October 2023, as the monoclonal antibody was not available for in-hospital immunisation of children born in September. However, the estimates from the present study suggest that immunisation of children born in September would have increased appreciably the number of RSV-related hospitalisations prevented, especially because this season was moved up. Extending the immunisation to infants born from April to August 2023 would have avoided additional RSV-related hospitalisations; however, the efficiency of this extension would have been notably lower, the program would have required a catch-up strategy, and the immunisation coverage would probably have been lower [17,23].

The number of hospitalisations for RSV prevented by nirsevimab immunisation of infants born in January 2024 could not been estimated in this early RSV season. According to historical data, the RSV epidemic in infants usually peaks in December or January and the number of hospitalisations for RSV is high in infants born in January [6]. Although this background supports the immunisation of children born in January, the weekly prospective revision of the updated RSV epidemiological surveillance may efficiently adapt the decision to end prospective immunisation of newborn children to the characteristics of each specific RSV epidemic season.

This study evaluated the nirsevimab effectiveness and the impact of a usage strategy against RSV-related hospitalisations. Since the benefit of this immunoprophylaxis could be observed in other RSV outcomes, such as emergency room and primary healthcare consultations, the total benefit of nirsevimab will be greater than that reported in the present study.

The results of this study offer a good reference to compare the effectiveness between passive immunisation of children and pregnant women vaccination with new RSV vaccines for preventing RSV disease in newborn children [24]. The results of the present study may be useful for economic studies comparing different strategies of use of nirsevimab. The prospective immunisation strategy evaluated in this study was highly efficient, because few nirsevimab doses were sufficient to prevent one hospitalisation. Since the cost of the immunoprophylaxis may be compensated by savings in health expenditures due to hospitalisation, this strategy could also be of interest for middle- and low-income countries [25]. Despite of the high effectiveness of the nirsevimab immunoprophylaxis, the high cost of this product makes an economic evaluation advisable to determine the optimal strategy of use in each country, and in particular, the introduction of the catch-up strategy.

The strengths of this study are the prospective recruitment of children at birth who were followed for weeks during periods of high RSV circulation, the use of registered information covering the whole population of the region, and the inclusion of only laboratory-confirmed cases. The study evaluates the effectiveness of nirsevimab and a possible strategy for its use in the population, providing valuable information for decision-makers in immunisation programs. This study used high-quality data from enhanced surveillance of hospitalisations for RSV in a region where RSV infection was routinely tested for in all infants admitted to the hospital for lower respiratory tract symptoms and all confirmed cases were revised by doctors to establish whether the admission was related to RSV infection.

The low number of children whose parents refused the immunisation limited the statistical power of this study, although the final number was sufficient to obtain precise estimates. Although comorbidities and prematurity could bias estimates, these variables were unavailable for all newborn children and could not be controlled for in the analysis. We cannot rule out an association between paternal refusal of immunisation and the risk of RSV-related hospitalisation; however, no case hospitalised for RSV had had a diagnosis of a high-risk condition prior to hospital admission. In Navarre, nirsevimab was also offered to all children aged less than 24 months with high-risk conditions [5,8]; however, there was no overlap with infants included in the present study. Estimates of the potential impact of immunoprophylaxis were obtained considering the immunisation coverage and the epidemiological pattern observed in the 2023–2024 seasons. Changes in these parameters could modify the impact of the intervention in coming years. Although the distribution of the infant hospitalisations for RSV by birth month followed a stable pattern over pre-pandemic seasons [6], some variability is possible [15,16]. Nevertheless, the extension of the immunoprophylaxis to children born in September and January covers a high percentage of infants of birth months with a higher risk of hospitalisation for RSV in Navarre and other sites, regardless of the annual variability [6,15,16]. While infant patients hospitalised for acute respiratory illness were routinely tested for RSV, other infant patients seen in the emergency room were tested occasionally; therefore, we cannot rule out bias in the analysis of emergency room patients, although this result was consistent with that referred to hospital admissions. Although this study was carried out in a specific region and season, nirsevimab effectiveness estimates, rates of prevented hospitalisations, and the number of infants who need to be immunised to prevent one RSV-related hospitalisation may be valid for other similar scenarios.

## 5. Conclusions

Nirsevimab prophylaxis in children at birth was highly effective at preventing hospitalisations for RSV-associated lower respiratory tract disease for several weeks after its administration. In regions where the peak of infant hospitalisation for RSV is expected in December or January, prospective immunisation at birth of children born from September to January may be a very efficient strategy for preventing a considerable proportion of hospitalisations for RSV in infants and may reduce the likelihood of paediatric hospital resources being overwhelmed during the weeks with high RSV circulation. The high cost of nirsevimab makes an economic evaluation advisable to conclude on the optimal use strategy in each country.

## Figures and Tables

**Table 1:** Crude and adjusted hazard ratios of the respiratory syncytial virus outcomes by nirsevimab immunisation status in the infants born in Navarre from October to December 2023 and follow-up until 28 January 2024.

	Infants *n*	Person Days	Cases *n*	Risk %	Rate per 1000 Person Days	Crude Hazard Ratio	95% Confidence Interval	Adjusted Hazard Ratio *	95% Confidence Interval
**Hospitalisation**									
Total									
Non-immunised	94	9321	8	8.51	0.86	1		1	
Immunised	1083	77,647	8	0.74	0.10	0.123	0.046–0.330	0.113	0.042–0.304
Male									
Non-immunised	49	4641	5	10.20	1.08	1		1	
Immunised	583	41,055	4	0.69	0.10	0.096	0.026–0.357	0.085	0.022–0.332
Female									
Non-immunised	45	4680	3	6.67	0.64	1		1	
Immunised	500	36,592	4	0.80	0.11	0.171	0.038–0.770	0.185	0.040–0.850
Infants born in October									
Non-immunised	33	4190	3	9.09	0.72	1		1	
Immunised	361	36,399	3	0.83	0.08	0.112	0.023–0.555	0.118	0.023–0.598
Infants born in November									
Non-immunised	35	3523	4	11.43	1.14	1		1	
Immunised	396	27,745	3	0.76	0.11	0.085	0.019–0.382	0.084	0.018–0.380
Infants born in December									
Non-immunised	26	1608	1	3.85	0.62	1		1	
Immunised	326	13,503	2	0.61	0.15	0.241	0.022–2.677	0.194	0.015–2.430
**Emergency-room attended**									
Non-immunised	94	9321	9	9.57	0.97	1		1	
Immunised	1083	77,647	11	1.02	0.14	0.141	0.058–0.341	0.121	0.049–0.297
**Intensive care unit admission**									
Non-immunised	94	9321	2	2.13	0.21	1		1	
Immunised	1083	77,647	3	0.28	0.04	0.203	0.034–1.224	0.141	0.023–0.868

* Result of Cox regression model using the calendar day as the underlying time scale, date of birth as entry time and adjusted for sex.

**Table 2:** Estimated impact of nirsevimab immunoprophylaxis in different birth cohorts according to the results observed in the 2023–2024 season in Navarre.

Birth Cohorts	RSV-Related Hospitalisations in the Absence of Immunoprophylaxis		Suggested Moment and Site of Administration	Immunisation Coverage with Nirsevimab *	Estimated Rate of RSV-Related Hospitalisations That Were Prevented or Preventable		Immunisations to Prevent One RSV-Related Hospitalisation
	*N* (%)	Rate per 1000 Person Days		%	*N* (%)	Rate per 1000 Person Days	*N*
**Immunised cohorts in Navarre in 2023–2024**							
October–December 2023	86.8 (56.8)	73.7	Just after birth in the hospital	92.0	70.8 (59.4)	60.2	15.3
January 2024 **	4 (2.6)	11.4	Just after birth in the hospital	92.0	4 (3.4)	11.4	80.5
**Other birth cohorts of interest**							
September 2023	24 (15.7)	60.0	Just after birth in the hospital	92.0	17.0 (14.3)	49.0	18.8
April–August 2023	29 (19.0)	15.7	Catch-up in primary healthcare	81.4	20.9 (17.5)	11.3	71.7
January–March 2023	9 (5.9)	8.8	Catch-up in primary healthcare	81.4	6.5 (5.5)	6.3	128.8
February–March 2024	0 (0)	0	Just after birth in the hospital	92.0	0 (0)	0	>600

RSV—respiratory syncytial virus. * The observed immunisation coverage of newborn children in Navarre (92.0%) was assumed for other birth cohorts that had been immunised in maternity wards. For the catch-up in primary healthcare, we used the immunisation coverage reported by Martinón-Torres et al. (81.4%) [17]. ** No RSV-related hospitalisation was observed among children born in January in the context of low circulation of RSV in the population. We have assigned an estimated number of hospitalisations prevented by nirsevimab immunoprophylaxis compatible with no immunoprophylaxis failures.

## Data Availability

The data presented in this study are available on request from the corresponding author.
